# Causal relationship between 412 gut microbiota, 1,400 blood metabolites, and diabetic nephropathy: a randomized Mendelian study

**DOI:** 10.3389/fendo.2024.1450428

**Published:** 2025-01-17

**Authors:** Bo-Ning Cao, Cai-Yan Zhang, Zhen Wang, Yao-Xian Wang

**Affiliations:** ^1^ Endocrinology Department, Dongzhimen Hospital, Beijing University of Chinese Medicine, Beijing, China; ^2^ General Surgery Department, The General Hospital of The People's Liberation Army, Beijing, China; ^3^ Administrative Department, Renal Research Institution of Beijing University of Chinese Medicine, Beijing, China

**Keywords:** Mendelian two-way randomization analysis, gut microbiota, diabetic nephropathy, probiotics, blood metabolites

## Abstract

**Objective:**

The aim of this study was to investigate the causal relationship between microbiota, diabetic nephropathy, and blood metabolites through a randomized Mendelian study.

**Methods:**

In this study, we used 412 microbiota as exposures, 1,400 blood metabolites as intermediaries, and diabetic nephropathy as the outcome. We conducted a two-way Mendelian randomization (MR) analysis to explore the causal relationship between microbiota and diabetic nephropathy, followed by mediation analyses and two-step MR to identify potential blood metabolites.

**Results:**

There is a causal relationship between microbiota and diabetic nephropathy. Specific bacteria and metabolites, such as *Escherichia coli str. K-12 substr. MG1655*, *Listeria monocytogenes 10403S*, *g_Adlercreutzia*, *g_Haemophilus*, *g_Bacteroides*, and *Escherichia coli CFT073*, and metabolites like pyrraline, glycocholenate sulfate, alpha-ketoglutarate, tetradecadienoate (14:2), Cys-gly oxidized, methylsuccinate, and various others, were identified. *Escherichia coli str. K-12 substr. MG1655* is positively related to alpha-ketoglutarate levels, while alpha-ketoglutarate levels and *Sphingomyelin (d18:1/18:1, d18:2/18:0)* are negatively related. The bacterial microbiota involved in fatty acid oxidation is associated with diabetic kidney disease (DKD) progression, positively correlated with glycocholenate sulfate levels, and negatively correlated with the phosphate linoleyl-tetraenyl-glycerol (18:2 to 20:4) ratio. Additionally, *Listeria monocytogenes 10403S* is positively correlated with N-acetyl-isoputreanine and negatively correlated with X-12462. Anaerobic fermentation-related bacteria were positively related to N-acetylcarnitine and 5-acetylamino-6-formyluracil and negatively correlated with 5-acetamino-6-amino-3-methyluracil (X-24243). *Escherichia coli CFT073* was positively associated with X-16580. Interactions between *Bacillus* species and metabolites such as d18:1/18:1, d18:2/18:0, 2-aminophenol sulfate, and cholate were negative when compared to tetradecadienoate (14:2). *g_Adlercreutzia* is positively correlated with N-delta-acetylornithine, methylsuccinate, and N-acetyl-isoputreanine but negatively correlated with N-acetylglucosamine and N-acetylgalactosamine. *g_Haemophilus* was positively associated with arachidoylcarnitine but negatively correlated with X-24531. The results were heterogeneous and multi-efficacious.

**Conclusions:**

For the first time, MR analysis provides supportive evidence for a bidirectional causal relationship between microbiota and diabetic nephropathy and identifies specific genes associated with the disease. The results suggest that probiotic therapy may play a significant role in preventing diabetic nephropathy and improving the quality of life and survival rates of affected patients. Furthermore, this study provides additional evidence of a causal relationship between specific microbiota, diabetic nephropathy, and blood metabolites.

## 1 Introduction

Diabetic nephropathy (DN) refers to kidney damage caused by diabetes and is one of the most common microvascular complications of the disease. Measuring urinary albumin levels and glomerular filtration rates (GFRs) is an important diagnostic measure (1) Type 2 diabetes is a rapidly growing global health issue, particularly with the aging population. The incidence of diabetes has been increasing annually, reaching 10.5% in 2021 (2), and it is projected that 579 million people will develop diabetes by 2045 (3). DN typically develops approximately 10 years after the onset of diabetes, with approximately 30%–40% of newly diagnosed diabetics developing the condition each year. Of these, approximately 30% progress to end-stage kidney disease (4). Diabetes with kidney complications significantly increases the risk of mortality compared to diabetes without kidney involvement (5, 6). As diabetes progresses, the prevalence of DN has steadily increased (7). Considerable progress has been made in understanding the pathogenesis of DN, particularly in its prevention and treatment (8, 9). Therefore, it is crucial to further elucidate the molecular mechanisms underlying DN and explore the processes of renal fibrosis.

Recent studies have identified a link between intestinal microbiota disorders and kidney disease. Clinical evidence suggests that imbalances in the gut microbiota may play a key pathological role in DN (10). The human intestinal microbiota is predominantly composed of *Firmicutes* and *Bacteroidetes* (11), which serve as potential diagnostic markers for microbial dysbiosis. In DN, the abundance of *Firmicutes* decreases, while that of *Bacteroidetes* increases, which is associated with impaired glucose tolerance and insulin resistance (12). Proteinuria is a critical marker in the early diagnosis of DN, and studies have shown increased levels of *Lactobacillus, Enterobacteriaceae*, and *Streptococcus* in these patients (13). Previous research suggests that gut microbiota may influence DN through its effects on blood metabolites (14). Short-chain fatty acids (SCFAs) produced by gut bacteria play a crucial role in regulating inflammatory and immune responses. An increased abundance of SCFA-producing bacteria can shift the intestinal environment toward an inflammatory state, contributing to tubular injury (15). In DN-induced tubular interstitial damage, bacteria such as *Actinobacterium, Ruminococcus*, and *Rikenella* are decreased, while the abundance of *Lactobacillus* and *Phascolarctobacterium* acetate significantly increases (16). Fecal transplantation and modulation of the intestinal microbiota in DN have been shown to reduce tubular interstitial damage by improving cholesterol homeostasis (17). Thus, gut microbiota and blood metabolites play a critical role in the progression of DN (18). This study aims to clarify the causal relationship between intestinal microbiota, blood metabolites, and DN using Mendelian randomization (MR).

MR has emerged as a widely used epidemiological analysis method in recent years (19). By leveraging the principle of randomized allele distribution during meiosis, it mitigates confounding factors and reduces the impact of reverse causation commonly seen in observational epidemiology (20). Genetic variants serve as instrumental variables (IVs) to assess causal relationships between exposure factors and outcomes (21). Two-sample bi-directional MR uses two separate genome-wide association study (GWAS) datasets to evaluate causal relationships between exposure and outcome, thereby enhancing the statistical power of the analysis (22).

To date, no MR analysis has been published on the bidirectional causal relationship between intestinal microbiota and DN. However, there is increasing evidence supporting the value of human genetic data in clinical studies of gut microbial features, enabling the use of MR to infer causal relationships between gut microbiota and DN (23). This study investigates the potential causal links between intestinal microbiota, blood metabolites, and DN by performing a bidirectional MR analysis using the latest genome-wide association data.

## 2 Materials and methods

### 2.1 Study design

**Figure 1:**
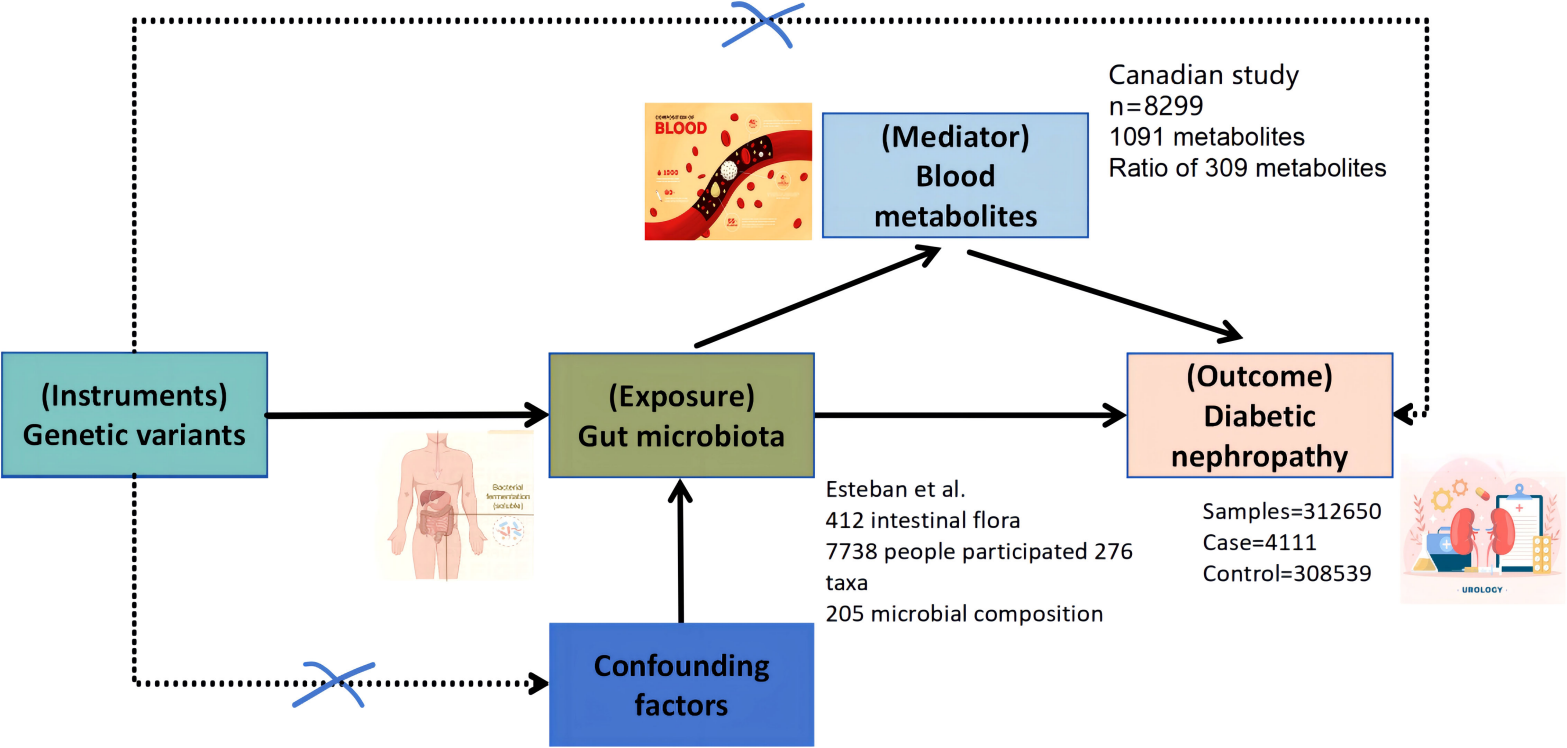
Bi-directional and intermediate Mendelian randomization analysis and hypothesis. Firstly, the causal relationship between gut microbiota and diabetic nephropathy was analyzed by two-sample bi-directional MR. Secondly, 1,400 blood metabolites were selected for subsequent randomized Mendelian analysis. Finally, a two-step MR analysis was performed to identify potentially mediated blood metabolites (the first step in screening blood metabolites associated with diabetic nephropathy). The second step is to further screen the gut microbiota associated with diabetic nephropathy and finally establish an intermediary analysis of blood metabolites from the gut microbiome to diabetic nephropathy.

This study employed a two-sample bidirectional MR approach to investigate the potential causal relationships between gut microbiota and DN. The goal was to comprehensively assess both the direct and reverse causal effects of gut microbiota on DN and *vice versa*, as well as the mediating role of blood metabolites in this pathway. The workflow of this study is outlined in **Figure 1**. The study was divided into three major components:

Bidirectional causal analysis: We analyzed the bidirectional causal relationship between 412 gut microbiota taxa and DN.

Causal effect of blood metabolites: We evaluated the causal relationship between 1,400 blood metabolites and DN.

Mediation analysis: We explored whether blood metabolites mediate the relationship between gut microbiota and DN.

The validity of the MR analysis is based on three key assumptions: The IVs are strongly associated with the exposure (gut microbiota or blood metabolites). The IVs are independent of confounding factors that affect both the exposure and the outcome. The IVs influence the outcome only through the exposure, with no pleiotropic effects.

The data on DN outcomes are derived from the Finnish database, which satisfies these assumptions. Ethical approval for the GWAS data used in this study has been obtained from the relevant ethics committees. A schematic of the study workflow is shown in **Figure 1**.

### 2.2 Sources of Exposure and Outcome Data

#### 2.2.1 Gut microbiota and blood metabolites

Gut microbiota data were obtained from a GWAS conducted by Esteban et al. (24), which included 7,738 participants and identified 412 microbial taxa. The study provided data on 207 taxa and 205 pathways reflecting microbial composition and activity. Blood metabolite data were sourced from a study by Kettunen et al. (25), which included 8,299 participants and identified 1,400 metabolites.

#### 2.2.2 Diabetic nephropathy GWAS data

Summary statistics for DN were obtained from the FinnGen study (26), which includes 260,405 participants. The fifth data release includes 4,984 cases of DN and 255,421 controls. Cases were identified using a comprehensive set of diagnostic codes, in line with World Health Organization (WHO) recommendations (27).

#### 2.2.3 Quality control of instrumental variables

To identify appropriate IVs for gut microbiota and blood metabolites, we first selected significant single-nucleotide polymorphisms (SNPs) with a *p*-value threshold of <1e-05. For each exposure, we applied a linkage disequilibrium (LD) clumping threshold of clump_kb=10,000 and clump_r2 = 0.001 to ensure independence of SNPs. We excluded weak IVs with an *F*-statistic < 10 to avoid weak instrument bias (28, 29). The SNP data were extracted for chromosomal locations, effect alleles, effect allele frequencies (EAF), effect sizes (β), standard errors (SE), and *p*-values.

To exclude potential confounding factors, we used the *PhenoScannerV2* database to validate and exclude SNPs associated with confounders. This rigorous quality control ensures the robustness and reliability of our results.

### 2.3 Data analysis

#### 2.3.1 Preliminary analysis

Inverse variance weighting (IVW) was used as the primary method for identifying causal relationships between gut microbiota and DN. IVW is preferred due to its ability to minimize pleiotropy and bias, making it a reliable method for causal inference (30). Additionally, we employed complementary methods, including weighted median, MR-Egger, simple modal, and weighted modal approaches, to evaluate the consistency and robustness of the causal estimates.

The results from MR analyses are reported as odds ratios (ORs) with 95% confidence intervals (CIs). Statistical significance was determined by an IVW *p*-value < 0.05, with directionality consistent across methods. We applied Bonferroni correction for multiple testing, considering the large number of exposures and outcomes. To examine reverse causality, a reverse MR analysis was also performed.

#### 2.3.2 Mediation analysis

We conducted a mediation analysis to investigate whether blood metabolites mediate the relationship between gut microbiota and DN. The steps in the mediation analysis are outlined as follows:

Step 1: First, we determined whether gut microbiota (412 taxa) had a causal effect on blood metabolites (1,400 metabolites).
Step 2: We then assessed whether these metabolites served as intermediaries in the pathway between gut microbiota and DN.

Two MR methods were used for mediation analysis:

Two-stage Mendelian randomization (TSMR) (31): This approach assumes no interactions between exposures and mediators. We estimated the causal effect of gut microbiota on DN (β_1_) from univariate MR, the causal effect of blood metabolites on DN (β_2_), and the causal effect of microbiota on metabolites (α).

Multivariate Mendelian randomization (MVMR) (32): This method allows for the simultaneous estimation of both the direct and indirect causal effects. In MVMR, the controlled direct effect of gut microbiota on DN was estimated (β_1_), while the indirect effect through metabolites was represented as α × β_2_.

All IVW results were adjusted for multiple comparisons using the false discovery rate (FDR) method, with FDR *q*-values reported.

#### 2.3.3 Sensitivity analysis

We conducted a sensitivity analysis to assess the robustness of the causal estimates and ensure that the results were not influenced by pleiotropy or heterogeneity. The following tests were employed:

MR-Egger intercept: To detect the presence of directional pleiotropy.

Cochran’s *Q* test: To assess the heterogeneity of the IVs (*p* < 0.05).

MR-PRESSO (33): To identify and correct for outliers (SNPs) contributing to pleiotropy and heterogeneity. SNPs with a significance level of *p* < 0.05 were flagged as outliers (see **Table 1**).

**Table 1:** Mendelian randomization analyses of the causal effects between gut microbiota and blood metabolites.

Exposure	Outcome	Nsnp	Methods	Beta	SE	OR (95% CI)	*p*-value	Cochran *Q*	Heterogeneity *p*-value	MR-Egger intercept	Intercept *p*-value	MR-PRESSO *p*-value
*E.coli_MG1655*	Sphingomyelin (d18:1/18:1, d18:2/18:0)	9	MR Egger	0.31	0.27	1.37 (0.80–2.34)	0.29	5.939	0.547	−0.04;5	0.135	0.394
			IVW	0.14	0.07	0.87 (0.76–1.00)	0.04					
	Alpha-ketoglutaramate	9	MR Egger	0.41	0.29	0.67 (0.38–1.17)	0.2	7.23	0.512	0.057	0.08	0.536
			IVW	0.16	0.07	1.18 (1.03–1.34)	0.02					
	Sphingomyelin (d18:2/16:0, d18:1/16:1)	9	MR Egger	0.21	0.27	1.24 (0.72–2.12)	0.46	6.108	0.635	−0.036	0.217	0.661
			IVW	0.15	0.06	0.86 (0.76–0.98)	0.02					
*FAO*	Glycocholenate sulfate	7	MR Egger	0.14	0.23	1.15 (0.74–1.80)	0.57	1.945	0.925	0.003	0.92	0.933
			IVW	0.16	0.06	1.18 (1.04–1.33)	0.01					
	Phosphate to linoleoyl-arachidonoyl-glycerol (18:2 to 20:4) [1] ratio	7	MR Egger	0.09	0.24	0.91 (0.57–1.46)	0.72	2.014	0.918	−0.008	0.8	0.921
			IVW	0.15	0.06	0.86 (0.76–0.97)	0.02					
*E.coli_MG1655*	X-07765	11	MR Egger	0.02	0.16	0.98 (0.72–1.34)	0.91	6.875	0.737	−0.018	0.656	0.724
			IVW	0.09	0.04	0.87 (0.76–1.00)	0.01					
	N-acetyl-L-glutamine	11	MR Egger	0.15	0.16	1.16 (0.86–1.58)	0.36	5.317	0.869	−0.039	0.172	0.882
			IVW	0.07	0.04	0.93 (0.87–0.99)	0.03					
*non.oxidative*	5-acetylamino-6-amino-3-methyluracil		MR Egger	0.17	0.36	1.18 (0.58–2.41)	0.67	1.204	0.945	−0.001	0.975	0.944
		6	IVW	0.15	0.08	0.87 (0.76–1.00)	0.05					
	X-24243	6	MR Egger	0.34	0.38	0.71 (0.34–1.49)	0.42	4.548	0.473	0.019	0.647	0.549
			IVW	0.16	0.08	0.85 (0.73–1.00)	0.04					
	N-acetylarginine	6	MR Egger	0.24	0.36	1.28 (0.63–2.58)	0.53	4.207	0.52	−0.009	0.826	0.556
			IVW	0.16	0.08	1.18 (1.01–1.37)	0.03					
	5-acetylamino-6-formylamino-3-methyluracil	6	MR Egger	0.04	0.37	1.04 (0.50–2.17)	0.91	2.507	0.776	0.013	0.749	0.812
			IVW	1.17	0.08	1.18 (1.01–1.39)	0.04					
*E.coli_CFT073*	X-16580		MR Egger	0.13	0.16	0.88 (0.64–1.21)	0.46	5.715	0.573	0.037	0.208	0.603
		8	IVW	0.09	0.04	1.10 (1.01–1.19)	0.03					
*L.monocytogenes*	N-acetyl-isoputreanine	14	MR Egger	0.13	0.24	1.13 (0.70–1.82)	0.61	10.92	0.618	−0.008	0.955	0.637
			IVW	0.11	0.05	1.12 (1.01–1.24)	0.03					
	X-12462	14	MR Egger	0.52	0.25	0.59 (0.36–0.97)	0.06	12.779	0.465	0.038	0.139	0.494
			IVW	0.13	0.05	0.88 (0.79–0.97)	0.02					
*biosynthesis.II*	Sphingomyelin (d18:1/18:1, d18:2/18:0)	12	MR Egger	−0.04;	0.16	0.96 (0.70–1.31)	0.78	10.928	0.445	0.018	0.429	0.482
			IVW	0.08	0.04	1.09 (1.00–1.17)	0.04					
	2-aminophenol sulfate	12	MR Egger	0.29	0.17	1.34 (0.96–1.87)	0.11	8.772	0.643	−0.089	0.244	0.647
			IVW	0.09	0.04	1.10 (1.01–1.19)	0.03					
	Tetradecadienoate (14:2)	12	MR Egger	−0.35	0.16	0.71 (0.51–0.97)	0.06	11.869	0.374	0.037	0.128	0.4
			IVW	−0.09	0.04	0.92 (0.84–1.00)	0.04					
	Cholate	12	MR Egger	0.08	0.23	1.09 (0.70–1.70)	0.72	16.836	0.113	−0.004;	0.889	0.121
			IVW	0.12	0.05	1.12 (1.01–1.25)	0.03					
	X-24531	12	MR Egger	−0.85	0.19	1.18 (1.01–1.37)	0.21	8.946	0.627	0.225	0.399	0.653
			IVW	−0.09	0.05	1.18 (1.01–1.37)	0.05					
*novo.biosynthesis*	Sphingomyelin (d18:1/18:1, d18:2/18:0)	13	MR Egger	−0.06	0.26	0.94 (0.57–1.56)	0.82	19.307	0.081	−0.008	0.768	0.085
			IVW	−0.13	0.07	0.88 (0.77–1.00)	0.04					
	N-acetylglucosamine/n-acetylgalactosamine	13	MR Egger	−0.18	0.2	0.89 (0.60–1.32)	0.58	7.547	0.819	0	0.991	0.829
			IVW	−0.18	0.05	0.89 (0.80–0.99)	0.03					
	N-acetyl-isoputreanine	13	MR Egger	0.1	0.23	1.11 (0.71–1.72)	0.66	13.416	0.34	0.002	0.921	0.336
			IVW	0.12	0.06	1.13 (1.01–1.27)	0.03					
	N-delta-acetylornithine	13	MR Egger	−0.13	0.21	0.88 (0.58–1.32)	0.54	5.873	0.922	0.028	0.223	0.911
			IVW	0.13	0.06	1.13 (1.02–1.26)	0.02					
*g_Adlercreutzia*	Methylsuccinate	6	MR Egger	0.35	0.24	1.42 (0.88–2.28)	0.22	3.461	0.629	−0.088	0.447	0.661
			IVW	0.15	0.06	1.16 (1.03–1.31)	0.01					
*Equolifaciens* sp.	Methylsuccinate	6	MR Egger	0.34	0.24	1.41 (0.88–2.25)	0.23	3.48	0.626	−0.087	0.461	0.663
			IVW	0.15	0.06	1.16 (1.03–1.31)	0.01					
*g_Haemophilus*	Arachidoylcarnitine (C20)	6	MR Egger	−0.84;	0.32	0.79 (0.42–1.48)	0.5	1.975	0.853	0.068	0.346	0.861
			IVW	0.1	0.05	1.10 (1.01–1.21)	0.04					
	X-24531	6	MR Egger	0.17	0.36	1.19 (0.59–2.39)	0.65	5.078	0.406	−0.059	0.453	0.476
			IVW	−0.18	0.05	0.89 (0.80–0.98)	0.02					
*g_Bacteroides*	Pyrraline	6	IVW	0.18	0.05	1.20 (1.09–1.32)	0	1.518	0.911	0.027	0.34	0.922
	Cys-gly, oxidized	6	IVW	0.11	0.05	1.12 (1.02–1.22)	0.02	1.469	0.917	0.025	0.357	0.921
	X-24243	6	IVW	0.11	0.05	1.12 (1.02–1.24)	0.02	1.503	0.913	0.026	0.352	0.931

Beta, standard errors (SE), and *p*-values were obtained from the Mendelian randomization analysis. The heterogeneity test in the IVW method was performed using Cochran’s *Q* statistic. SNP, single-nucleotide polymorphism; Ph, *p*-value for heterogeneity; *p*_intercept_, *p*-value for the intercept of the MR-Egger regression; IVW, inverse-variance-weighted; MR, Mendelian randomization.

We also performed a leave-one-out analysis (34) to determine whether any single SNP disproportionately influenced the MR results (35).

Finally, we used a two-step randomization approach to explore the role of blood metabolites as mediators in the relationship between gut microbiota and DN. All analyses were conducted in the R Studio environment (version 4.3.1).

## 3 Results

### 3.1 Screening of instrumental variables

In this study, IVs were rigorously controlled in MR analysis to assess the causal relationship between 412 gut microbiota taxa and DN. Genetic variations with an *F*-statistic greater than 10 were considered strong IVs. Sensitivity analysis was conducted using the MR-Egger intercept and MR-PRESSO method to test for pleiotropy, and Cochran’s *Q* test (*p* < 0.05) was used to assess the heterogeneity of the IVs.

### 3.2 Two-sample and bidirectional Mendelian randomization analysis of gut microbiota and diabetic nephropathy

**Figure 2:**
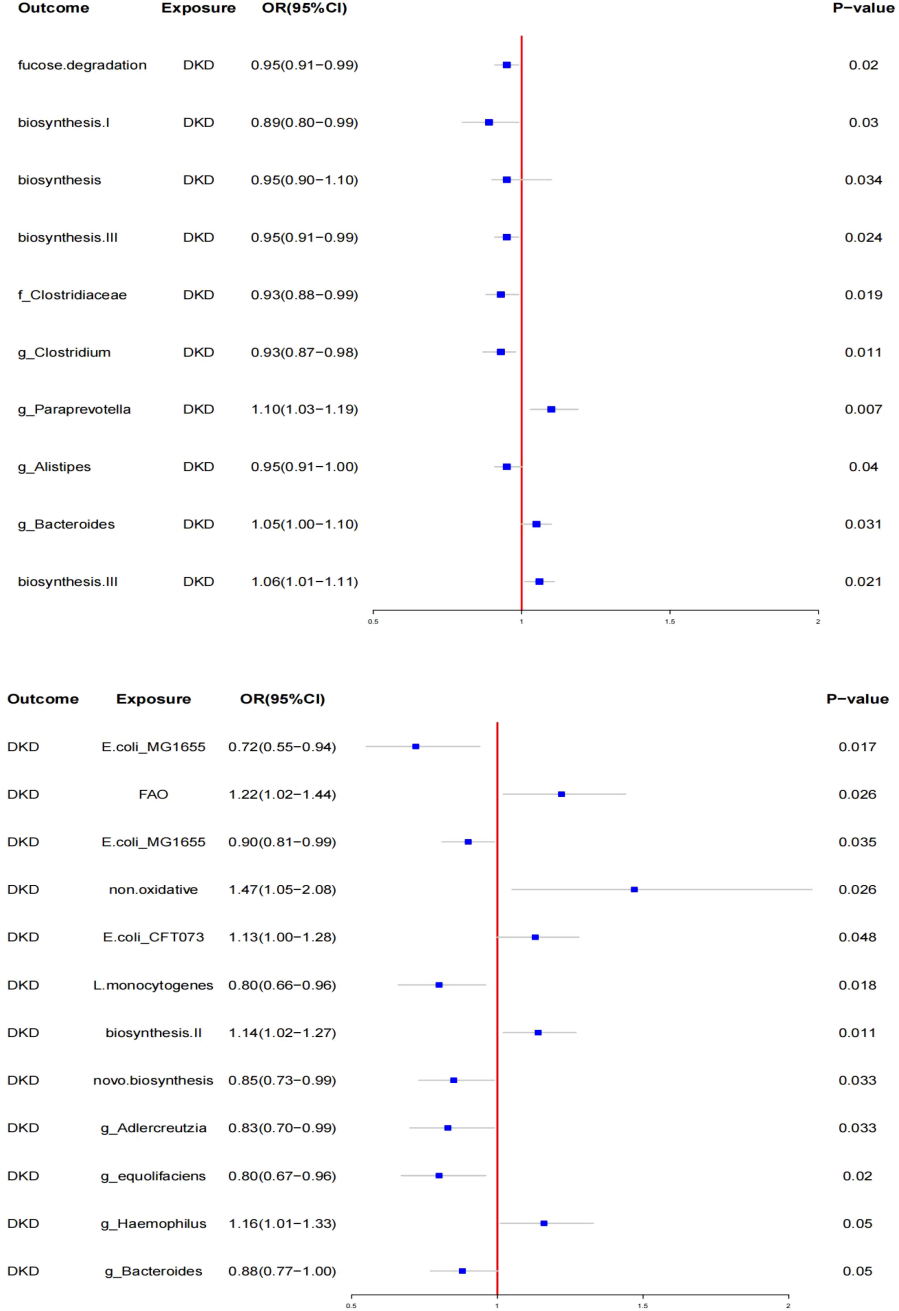
Mendelian randomization results of causal effects between gut microbiotas and DKD.

In the MR analysis of gut microbiota, SNPs associated with gut microbiota were used as IVs. The IVW method identified 12 specific gut microbiota taxa with significant causal effects on DN (see **Figure 2**).

#### 3.2.1 **Protective taxa**

- *Escherichia coli* str. K-12 substr. MG1655 series (OR: 0.72, 95% CI: 0.55–0.94, *p <* 0.05)
- *Listeria monocytogenes* 10403S (OR: 0.80, 95% CI: 0.66–0.96, *p <* 0.05)
- *E. coli* str. K-12 substr. MG1655 series (OR: 0.90, 95% CI: 0.81–0.99, *p <* 0.05)
- *Bacteria from guanosine nucleotides* (OR: 0.85, 95% CI: 0.73–0.99, *p <* 0.05)
- *Adlercreutzia* (OR: 0.83, 95% CI: 0.70–0.99, *p <* 0.05)
- *Adlercreutzia sp. Adlercreutzia equolifaciens* (OR: 0.80, 95% CI: 0.67–0.96, *p <* 0.05)
- *Haemophilus* (Paraemophilus) (OR: 0.88, 95% CI: 0.77–1.00, *p <* 0.050)
- *Bacteroides* (OR: 0.88, 95% CI: 0.77–1.00, *p <* 0.05)

#### 3.2.2 **Risk taxa**

- Fatty acids β (OR: 1.22, 95% CI: 1.01–1.44, *p <* 0.05)
- New findings (OR: 1.47, 95% CI: 1.05–2.00, *p <* 0.05), suggesting a significant association, but further investigation is needed due to potential issues with classification.
- *Escherichia coli CFT073* (OR: 1.13, 95% CI: 1.10–1.28, *p <* 0.05)

#### 3.2.3 Biosynthesis pathways

Biosynthesis II. Plants (OR: 1.14, 95% CI: 1.03–1.27, *p <* 0.05)

No significant pleiotropy or heterogeneity was found using the MR-Egger test and Cochran’s *Q* test. Reverse MR analysis revealed changes in the relative abundance of gut microbiota taxa after the onset of diabetic kidney disease (DKD). Specifically, the relative abundance of three taxa increased, while seven taxa showed a decrease (see **Figure 2**). The most significantly affected gut bacterial population in DKD was *Paraprevotella xylaniphila* (OR: 1.14, 95% CI: 1.02–1.27, *p <* 0.05). Sensitivity analysis confirmed the robustness of these findings.

### 3.3 Mediation analysis of potential blood metabolites and DKD

**Figure 3:**
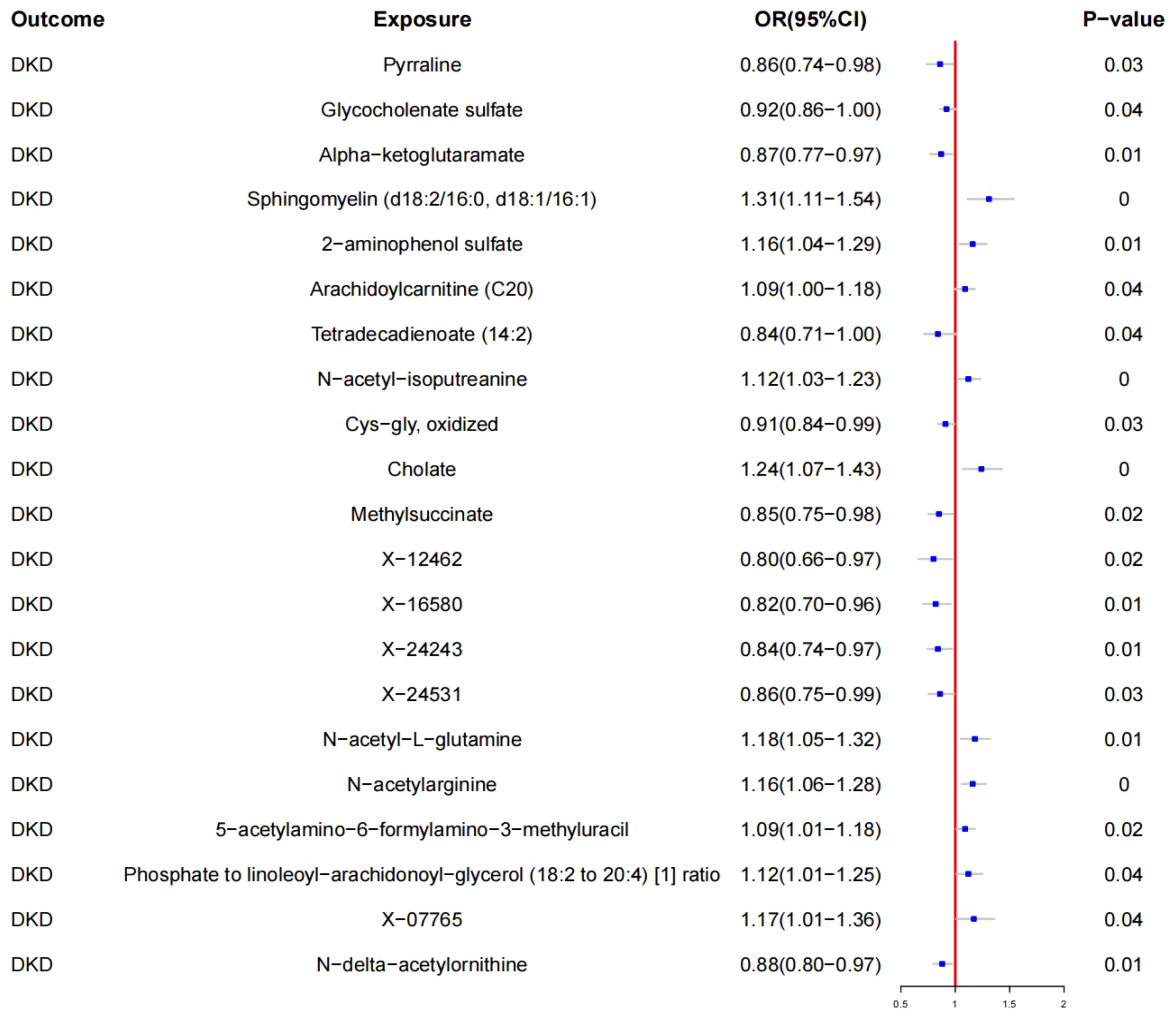
Mendelian randomization results of causal effects between Blood metabolites and DKD.

Using IVW, a causal relationship was found between 20 blood metabolites and DKD (see **Figure 3**, **Table 2**).

**Table 2:** Mendelian randomization analyses of the causal effects between blood metabolites and dkd.

Outcome	Nsnp	OR (95% CI)	*p*-value	Cochran *Q*	Heterogeneity *p*-value	MR-Egger intercept	Intercept *p*-value	MR-PRESSO *p*-value
Pyrraline	19	0.86 (0.74–0.98)	0.03	23.782	0.162	−0.017	0.322	0.165
Glycocholenate sulfate	29	0.92 (0.86–1.00)	0.04	38.453	0.090	−0.002	0.876	0.141
Alpha-ketoglutaramate	17	0.87 (0.77–0.97)	0.01	13.627	0.626	−0.004	0.773	0.69
Sphingomyelin (d18:2/16:0, d18:1/16:1)	17	1.31 (1.11–1.54)	0.00	15.416	0.494	0.007	0.704	0.532
2-aminophenol sulfate	31	1.16 (1.04–1.29)	0.01	35.058	0.241	−0.019	0.184	0.239
Arachidoylcarnitine (C20)	26	1.09 (1.00–1.18)	0.04	16.750	0.891	0.005	0.700	0.893
Tetradecadienoate (14:2)	11	0.84 (0.71–1.00)	0.04	7.252	0.701	−0.024	0.312	0.742
N-acetyl-isoputreanine	29	1.12 (1.03–1.23)	0.01	24.843	0.636	−0.011	0.405	0.637
Cys-gly, oxidized	20	0.91 (0.84–0.99)	0.03	15.604	0.683	0.010	0.388	0.633
Cholate	14	1.24 (1.07–1.43)	0.00	10.226	0.676	0.028	0.290	0.727
Methylsuccinate	19	0.85 (0.75–0.98)	0.02	12.168	0.838	−0.030	0.101	0.867
X-12462	9	0.80 (0.66–0.97)	0.02	7.206	0.515	−0.234	0.289	0.592
X-16580	14	0.82 (0.70–0.96)	0.01	11.391	0.578	0.024	0.437	0.604
X-24243	20	0.84 (0.74–0.97)	0.01	21.910	0.289	−0.030	0.150	0.3
X-24531	19	0.86 (0.75–0.99)	0.03	24.806	0.130	−0.023	0.340	0.11
N-acetyl-L-glutamine levels	15	1.18 (1.05–1.32)	0.01	26.470	0.023	−0.005	0.778	0.081
N-acetylarginine levels	21	1.16 (1.06–1.28)	0.00	28.053	0.108	0.004	0.771	0.166
5-Acetylamino-6-formylamino-3-methyluracil	19	1.09 (1.01–1.18)	0.02	19.654	0.353	−0.007	0.566	0.435
Phosphate to linoleoyl-arachidonoyl-glycerol (18:2 to 20:4) [1] ratio	21	1.12 (1.01–1.25)	0.04	26.520	0.149	0.018	0.272	0.154
X-07765	20	1.17 (1.01–1.36)	0.04	28.097	0.082	0.005	0.809	0.118
N-delta-acetylornithine	22	0.88 (0.80–0.97)	0.01	27.137	0.166	0.044	0.004	0.164

Odds ratios, 95% CI, and *p*-values were obtained from Mendelian randomization analysis. The heterogeneity test in the IVW method was performed using Cochran’s *Q* statistic. SNP, single-nucleotide polymorphism; CI, confidence interval; Ph, *p*-value for heterogeneity; *p*_intercept_, *p*-value for the intercept of the MR-Egger regression; IVW, inverse-variance-weighted; MR, Mendelian randomization.

#### 3.3.1 Protective metabolites

- Pyrraline (OR: 0.86, 95% CI: 0.74–0.98; *p <* 0.05)
- Glycocholenate sulfate (OR: 0.92, 95% CI: 0.86–1.00; *p <* 0.05)
- Alpha-ketoglutarate (OR: 0.87, 95% CI: 0.77–0.97; *p <* 0.05)
- Tetradecadienoate (14:2) (OR: 0.84, 95% CI: 0.71–1.00; *p <* 0.05)
- Cys-gly oxidized (OR: 0.91, 95% CI: 0.84–0.99; *p <* 0.05)
- Methylsuccinate (OR: 0.85, 95% CI: 0.75–0.98; *p <* 0.05)
- X-12462 (OR: 0.80, 95% CI: 0.66–0.97; *p <* 0.05)
- X-16580 (OR: 0.82, 95% CI: 0.70–0.96; *p <* 0.05)
- X-24243 (OR: 0.84, 95% CI: 0.74–0.97; *p <* 0.05)
- X-24531 (OR: 0.86, 95% CI: 0.75–0.99; *p <* 0.05)
- N-delta-acetylornithine (OR: 0.88, 95% CI: 0.80–0.97; *p <* 0.05)

#### 3.3.2 Risk metabolites

- Sphingomyelin (d18:2/16:0, d18:1/16:1) (OR: 1.31, 95% CI: 1.11–1.54; *p <* 0.05)
- 2-aminophenol sulfate (OR: 1.16, 95% CI: 1.04–1.29; *p <* 0.05)
- Arachidoylcarnitine (C20) (OR: 1.09, 95% CI: 1.00–1.18; *p <* 0.05)
- N-acetyl-isoputreanine (OR: 1.12, 95% CI: 1.03–1.23; *p <* 0.05)
- Cholate (OR: 1.24, 95% CI: 1.07–1.43; *p <* 0.05)
- N-acetyl-L-glutamine (OR: 1.18, 95% CI: 1.05–1.32; *p <* 0.05)
- N-acetylarginine (OR: 1.16, 95% CI: 1.06–1.28; *p <* 0.05)
- 5-acetylamino-6-formylamino-3-methyluracil (OR: 1.09, 95% CI: 1.01–1.18; *p <* 0.05)
- Phosphate to linoleoyl-arachidonoyl-glycerol (18:2 to 20:4) [1] ratio (OR: 1.12, 95% CI: 1.01–1.25; *p <* 0.05)
- X-07765 (OR: 1.17, 95% CI: 1.01–1.36; *p <* 0.05)

#### 3.3.3 Key findings

- *Escherichia coli* str. K-12 substr. MG1655 series (OR: 0.87, 95% CI: 0.78–0.97; *p <* 0.05)
- *Adlercreutzia* (OR: 0.83, 95% CI: 0.70–0.99; *p <* 0.05)
- Biosynthesis II. Plants (OR: 1.14, 95% CI: 1.03–1.27; *p <* 0.05)

By analyzing specific blood metabolites as intermediates, we identified that 11 protective gut microbiota-related metabolites reduced the risk of DN, while 10 risk metabolites significantly increased the risk.

### 3.4 Mendelian randomization analyses of the causal effects between gut microbiota and blood metabolites

We found that *Escherichia coli strain K-12 substrain MG1655* (OR: 0.87, 95% CI: 0.55–0.94, *p <* 0.05) serves as a protective factor against DKD. This strain increases alpha-ketoglutarate levels while lowering *s*phingomyelin (d18:1/18:1, d18:2/18:0) and sphingomyelin (d18:2/16:0, d18:1/16:1) levels. Gut microbiota associated with fatty acid oxidation (OR: 1.22, 95% CI: 1.02–1.44, *p <* 0.05) is linked to the progression of DKD, increasing glycocholenate sulfate levels and decreasing the phosphate to linoleoyl-arachidonoyl-glycerol (18:2 to 20:4) ratio, which is detrimental to DKD.

*Listeria monocytogenes 10403S* (OR: 0.90, 95% CI: 0.81–0.99, *p <* 0.05) exerts a protective effect on DKD by downregulating X-07765 and N-acetyl-l-glutamine levels. Additionally, *Listeria monocytogenes 10403S* (OR: 0.80, 95% CI: 0.66–0.96, *p <* 0.05) increases N-acetyl-isoputreanine levels and decreases X-12462 levels, which helps protect against DKD by lowering X-12462 content.

Anaerobic yeast-related gut microbiota (OR: 1.47, 95% CI: 1.05–2.08, *p <* 0.05) contributes to the development of DKD by increasing N-acetylarginine and 5-acetylamino-6-formylamino-3-methyluracil levels, while decreasing X-24243 levels*. Escherichia. coli CFT073* (OR: 1.13, 95% CI: 1.00–1.28, *p <* 0.05) is a harmful taxon for DKD, raising X-16580 levels. Similarly, the *Bacillus* phylum (OR: 1.14, 95% CI: 1.03–1.27, *p <* 0.05) is detrimental to DKD, as it increases sphingomyelin (d18:1/18:1, d18:2/18:0), 2-aminophenol sulfate, cholesterol, and X-24531 levels, while decreasing tetradecadienoate (14:2) levels.

Guanosine ribonucleotide biosynthesis-related gut microbiota (OR: 0.85, 95% CI: 0.73–0.99, *p <* 0.05) is beneficial and protective against DKD by increasing N-delta-acetylornithine and N-acetyl-isoputreanine levels, while decreasing sphingomyelin (d18:1/18:1, d18:2/18:0) and N-acetylglucosamine/N-acetylgalactosamine levels (see **Table 1**). *g_Adlercreutzia* (OR: 0.83, 95% CI: 0.70–0.99, *p <* 0.05) and g_Adlercreutzia.s_Adlercreutzia_equolifaciens (OR: 0.80, 95% CI: 0.67–0.96, *p <* 0.05) protect against DKD by increasing methylsuccinate levels. *g_Haemophilus* (OR: 0.88, 95% CI: 0.77–1.00, *p <* 0.05) and *g_Bacteroides* (OR: 0.88, 95% CI: 0.77–1.00, *p <* 0.05) also have protective effects, with *g_Haemophilus* increasing arachidoylcarnitine (C20) levels and decreasing X-24531 levels, demonstrating heterogeneous and multi-efficacious results.

We validated the mediating effects of blood metabolites identified by MVMR in TSMR. Focusing on the indirect effects and ratios mediated by two major classes of lipid metabolites, we found that sphingomyelin (d18:1/18:1, d18:2/18:0) and methylsuccinate remained significant after GM adjustment (**Table 3**). Overall, we observed indirect effects of sphingomyelin (d18:1/18:1,d18:2/18:0) and methylsuccinate between biosynthesis II*, g_Adlercreutzia*, and DN, with mediated proportions of 8.5% (*p <* 0.05) and 10.9% (*p <* 0.05), respectively (see **Table 3**).

**Table 3:** Multivariable Mendelian randomization analyses of the causal effects between gut microbiota, blood metabolites, and DKD.

Exposure	Mediator	Direct effect (β1* ± SE)	Direct effect (β2* ± SE)	Indirect effect (α×β2* ± SE)	*p*	Proportion mediated (α×β2*/β1)
*biosynthesis.II*	Sphingomyelin (d18:1/18:1, d18:2/18:0)	−0.33 ± 0.14	−0.20 ± 0.07	−0.028 ± 0.068	0.044	0.085
*g_Adlercreutzia*	Methylsuccinate	−0.22 ± 0.09	−0.16 ± 0.07	−0.024 ± 0.059	0.011	0.109

Beta (β), standard errors (SE), and *p*-values were obtained from multivariable Mendelian randomization analysis. β1* and β2* represent the controlled direct effects of each pair of bacteria and metabolite on DKD after adjusting for each other. α is the causal effect of exposure on mediator; indirect effect (α×β2*) is the effect of exposure on DKD via corresponding mediator; β1 is the total effect of exposure on DKD; proportion mediated is calculated as the “indirect effect/total effect.”

## 4 Discussion

Our research is innovative in utilizing MR to investigate the causal relationship between gut microbiota and DN (DKD), while also exploring the mediating role of blood metabolites in this connection. This study is unique in employing TSMR and MVMR to examine potential mediation by blood metabolites between gut microbiota and DKD. We identified 12 gut microbiota taxa causally linked to DKD, which, in turn, influence the relative abundance of 10 taxa. Through TSMR and MVMR as intermediary analyses, we discovered 13 blood metabolites associated with these 12 gut microbiota taxa and DKD.

In particular, *E. coli str. K-12 substr. MG1655* was found to reduce sphingomyelin levels by increasing α-ketoglutarate levels. Sphingomyelin levels (d18:2/16:0 and d18:1/16:1) were identified as protective against DKD. Moreover, our study highlighted the protective role of bacteria, such as *Lactobacillus* and Adler-Kreuzia, against DKD. Previous studies have shown that *Bacillus* spp. are involved in bile acid metabolism in DN, degrading lipopolysaccharides (LPS), inhibiting inflammation, and improving endotoxemia (36). However, other studies have indicated that Gram-negative bacteria, including *Bacteroidetes*, *Proteobacteria*, *Fusobacteria*, and *Verrucomicrobia*, are prevalent in DKD patients (37), leading to elevated *LPS* concentrations and the accumulation of inflammatory markers such as CRP, TNF-α, and IL-6. Clinical studies have also shown a decrease in the corresponding proportions of thick-walled mycobacteria and gut microbiota in DKD patients (38).

Our findings suggest that *E. coli* CFT073 and *Haemophilus parainfluenzae* are associated with the progression of DKD, potentially through other mechanisms, rather than fatty acid oxidation or anaerobic enzyme activity (12). Studies have shown that fatty acid β-oxidation can activate endoplasmic reticulum stress and excessive production of reactive oxygen species (ROS), leading to cellular dysfunction and contributing to the onset of DKD (39).

MR analysis confirmed the impact of several blood metabolites on the causal relationship between gut microbiota and DKD. Unlike commonly accepted biomarkers such as trimethylamine N-oxide (TMAO) and SCFAs, our study emphasizes the causal link between lipid metabolism and DKD. Our MR analysis demonstrated that sphingomyelin (d18:2/16:0, d18:1/16:1) and cholesterol levels are positively correlated with the risk of DKD progression. Sphingomyelins are crucial for glomerular and endothelial function. Lipidomic analysis has shown that sphingomyelins and phosphatidylcholine are associated with renal dysfunction and all-cause mortality in type 1 diabetes (40). Another MR analysis indicated that various lipoproteins protect against DKD (41).

Our mediation analysis provides a genetic basis for the causal relationship between gut microbiota and DKD. We found that *E. coli, Bacillus*, and *Adlercreutzia* are directly related to lipid metabolism, involving plasma sheath phospholipids and other metabolites. Furthermore, amino acids like α-ketoglutarate, isoleucine, and cysteine-glycine serve as intermediaries in the causal relationship between gut microbiota and DKD. Zhu (2022) suggested that amino acid metabolism plays a crucial role in the progression of diabetes mellitus (DM) and DN, with high levels of L-leucine and isoleucine significantly associated with a rapid decline in GFR.

Previous studies have not directly linked *E. coli, Bacillus*, and *Adlercreutzia* to lipid and amino acid metabolism. However, Han et al. studied the treatment of DKD with Yi kidney granules, identifying lactobacilli as positively related to sphingosine in sphingolipid metabolism and L-tyrosine in phenylalanine, tyrosine, and tryptophan biosynthesis (42). Our MR study confirmed a positive correlation between isoleucine and DKD, as well as between 2-aminophenol sulfate, cholate levels, and DKD progression. Uremic toxins, such as indoxyl sulfate and p-cresol sulfate, are closely related to CKD development, with imbalances in gut microbiota accelerating toxin production (43). Excessive levels of gut microbiota metabolites, such as 2-aminophenol sulfate and cholates, exacerbate kidney damage in DKD (44). The metabolism of choline, carnitine, and phosphatidylcholine by gut microbiota forms TMAO, which is then oxidized in the liver, highlighting the causal relationship between gut microbiota and lipid metabolism regulation (45).

The primary strength of this study lies in its comprehensive analysis of 412 gut microbiota taxa and 1,400 blood metabolites in relation to DKD. The study included a large sample size, utilizing data from 7,738 individuals for gut microbiota and 8,299 individuals for blood metabolites. This extensive dataset enabled us to explore the causal relationships using robust MR techniques.

However, this study has several limitations. Firstly, the findings predominantly apply to individuals of European descent, which may limit their generalizability to other ethnic groups. Differences in lifestyle, host metabolism, and gut microbiota composition across populations necessitate caution when interpreting these results for non-European groups. Future research should include more diverse populations to improve the generalizability of the findings. Additionally, despite rigorous efforts to identify and account for IV anomalies, potential pleiotropic effects may still exist. MR analysis is hypothesis-driven, and establishing a causal relationship between gut microbiota and DKD requires further experimental and clinical research.

## 5 Conclusion

This study provides robust evidence of a causal relationship between gut microbiota and DN, mediated by specific blood metabolites. These findings highlight the potential of gut microbiota and blood metabolites as novel biomarkers and therapeutic targets for DN. Probiotic therapy could significantly improve the quality of life and survival rates for patients with diabetic nephropathy.

## Data Availability

The original contributions presented in the study are included in the article/**Supplementary Material**. Further inquiries can be directed to the corresponding authors.
